# N-Cadherin in Neuroblastoma Disease: Expression and Clinical Significance

**DOI:** 10.1371/journal.pone.0031206

**Published:** 2012-02-15

**Authors:** Tim Lammens, Katrien Swerts, Lara Derycke, Annemie De Craemer, Sara De Brouwer, Katleen De Preter, Nadine Van Roy, Jo Vandesompele, Frank Speleman, Jan Philippé, Yves Benoit, Klaus Beiske, Marc Bracke, Geneviève Laureys

**Affiliations:** 1 Department of Pediatric Hematology-Oncology, Ghent University Hospital, Ghent, Belgium; 2 Department of Radiation Oncology and Experimental Cancer Research, Ghent University Hospital, Ghent, Belgium; 3 Center for Medical Genetics, Ghent University Hospital, Ghent, Belgium; 4 Department of Clinical Chemistry, Microbiology and Immunology, Ghent University Hospital, Ghent, Belgium; 5 Department of Pathology, Rikshospitalet, Oslo, Norway; Ghent University, Belgium

## Abstract

One of the first and most important steps in the metastatic cascade is the loss of cell-cell and cell-matrix interactions. N-cadherin, a crucial mediator of homotypic and heterotypic cell-cell interactions, might play a central role in the metastasis of neuroblastoma (NB), a solid tumor of neuroectodermal origin. Using Reverse Transcription Quantitative PCR (RT-qPCR), Western blot, immunocytochemistry and Tissue MicroArrays (TMA) we demonstrate the expression of N-cadherin in neuroblastoma tumors and cell lines. All neuroblastic tumors (n = 356) and cell lines (n = 10) expressed various levels of the adhesion protein. The N-cadherin mRNA expression was significantly lower in tumor samples from patients suffering metastatic disease. Treatment of NB cell lines with the N-cadherin blocking peptide ADH-1 (Exherin, Adherex Technologies Inc.), strongly inhibited tumor cell proliferation *in vitro* by inducing apoptosis. Our results suggest that N-cadherin signaling may play a role in neuroblastoma disease, marking involvement of metastasis and determining neuroblastoma cell viability.

## Introduction

Neuroblastoma (NB) is the most common extracranial solid tumor in children and accounts for 8–10% of all childhood malignancies [1], [2]. This neoplasm consists of primitive neuroblasts, derived from neural crest cells or sympathogonia. Approximately 40% of all NB patients suffer metastatic disease at diagnosis. Despite multimodal therapy, these children have a poor clinical outcome (5-year survival rate of 30% to 40%) [1]. Although our understanding of the heterogeneous nature of primary neuroblastoma has significantly improved, the metastatic process, often responsible for the unfavorable outcome, remains ill understood [3]–[6]. Studies on metastatic disease provide evidence that during this multistep process, loss of adhesion seems to be a crucial factor [7]–[9]. It has been shown that cell-cell adhesion molecules such as cadherins play a crucial role during the metastatic process. Consequently, those cell-cell interaction proteins form an interesting target for anti-tumor therapy [10]–[12].

In humans, the cadherin superfamily of adhesion molecules consists of more than 80 members. However, the most extensively studied are epithelial (E-) cadherin and neural (N-) cadherin. Whereas E-cadherin is mainly found in epithelial cells, promoting tight cell-cell associations, N-cadherin (CDH2) is primarily found in neuronal tissues and fibroblasts. Noteworthy, N-cadherin expression changes are crucial for correct migration of the neural crest cells during early embryonic development. Downregulation of N-cadherin on these cells is essential to allow migration away from the neural tube and contributes to the formation of a diverse array of tissues such as the peripheral nervous system, melanocytes, craniofacial cartilage and bone [13]. Members of the N-cadherin family are characterized by a large extracellular domain which mediates calcium dependent homophilic interaction between cadherins, expressed by neighboring cells [14]. In addition, interactions between cadherins and other receptors such as Fibroblast Growth Factor Receptor (FGFR) have been described [11], [15]. Through their highly conserved cytoplasmatic tail, cadherins bind catenins, linking them to the actin-based cytoskeleton [16].

It is generally accepted that metastasis is preceded by the loss of E-cadherin mediated cell-cell adhesion [17], [18]. The loss of E-cadherin is often accompanied by *de novo* expression of N-cadherin, promoting cell motility and migration. Together, these observations have been denominated ‘the cadherin switch’. In addition to promoting motility and migration, N-cadherin homophilic interaction between tumor cells and surrounding tissue (e.g. stroma, endothelium) has been shown to facilitate the transit and survival of tumor cells in distant organs [19]–[21]. Thus, N-cadherin might be an ideal drug target as inhibition might prevent tumor metastasis [22]. Examples of invasive tumors where N-cadherin is downregulated have also been identified. In osteosarcoma, N-cadherin inhibits cell migration and the formation of metastasis [23], [24]. Similarly, in ovarian carcinoma, N-cadherin is expressed during different stages, although, one report mentioned that mucinous cystadenomas are N-cadherin negative [25].

ADH-1 (Exherin, Adherex Technologies Inc.) is a novel cyclic pentapeptide which contains a cell adhesion recognition site (His-Ala-Val) important for N-cadherin interaction. Previous reports showed that peptides containing this sequence disrupt cell adhesion, induce apoptosis, and alter the intracellular distribution of β-catenin and actin in endothelial cells. Moreover, ADH-1 has been evaluated as an anti-tumor agent *in vivo* in phase I clinical trials [26]–[29].

In this study, we tried to unravel the role of N-cadherin in the metastatic process of neuroblastoma cells by examining the N-cadherin gene and protein expression in NB cell lines and primary tumors. N-cadherin expression was confirmed in all tumors and cell lines. Interestingly, low N-cadherin expression was significantly associated with metastatic disease. Inhibition of the N-cadherin function by the specific peptide inhibitor ADH-1 strongly provoked apoptosis of NB cell lines.

## Materials and Methods

### Patients and cell lines

The patient samples analyzed by RT-qPCR, represent a selection of the sample set previously described by Vermeulen *et al.* [30]. Patient characteristics are summarized in Table 1. The NB patients were diagnosed and staged according to the International Neuroblastoma Staging System (INSS) [31]. The ethical committee (Ethical Committee Ghent University Hospital) approved the study and written informed consent was obtained from the patients and/or their parents.

**Table 1:** Summary of patient characteristics.

	qPCR cohort(n = 356)	TMA cohort(n = 84)*
**Age (months)**		
Median (range)	10.7 (0–163)	16 (0–153)
**Gender**		
Male	52% (185)	44% (37)
Female	48% (171)	56% (47)
**MYCN status**		
Not amplified	85% (302)	75% (63)
Amplified	13% (47)	13% (11)
Duplicated	2% (7)	1% (1)
**INSS stage**		
Stage 1	35% (124)	23% (19)
Stage 2	16% (58)	17% (14)
Stage 3	17% (59)	11% (9)
Stage 4	22% (80)	29% (24)
Stage 4S	10% (35)	5% (4)
**Follow-up**		
Relapse/progression	35% (126)	40% (34)
CCR	65% (230)	60% (50)

*Remark that not for all patients included in the TMA cohort complete information was available.

Ten NB cell lines (CLB-GA, IMR32, SH-SY5Y, SK-N-BE(1n), SK-N-BE(2c), SK-N-FI, STA-NB-10, SJ-NB-10, SHEP and SK-N-SH) were included in this study (See Table S1). As controls, HCA2-hTERT fibroblasts (kindly provided by C. Jones, Cardiff University, UK) and MCF-7 epithelial cell cultures (ACC115, DSMZ, Germany) were used. Cells were cultured in RPMI 1640 medium supplemented with 15% fetal calf serum, 2 mM glutamine, 100 IU/mL penicillin, and 100 µg/mL streptomycin (medium and supplements from Gibco–Invitrogen, Merelbeke, Belgium) in a humidified atmosphere with 5% CO_2_ at 37°C.

### Immunocytochemistry

Cytospins were immunocytochemically stained as described previously [32]. Briefly, large diameter (17 mm) cytospins containing 5×10^5^ cells were incubated with an unlabeled mouse monoclonal anti-human N-cadherin antibody (clone 3B9, Invitrogen, Merelbeke, Belgium). Controls where the primary antibody was replaced by a non-related IgG1 antibody (Dako Corporation, Glostrup, Denmark company) were included to evaluate the background staining. During a second and third incubation step, cells were incubated with an unlabeled rabbit anti-mouse antibody (Dako Corporation) and APAAP complexes (Dako Corporation), respectively. The bound alkaline phosphatase complexes were stained using the Dako Fuchsin+™ Substrate Chromogene System (Dako Corporation). Cells were counterstained with hematoxylin and examined under a light microscope.

### Viability assay

Cells were seeded in triplicate in a 96-well plate (200,000 cells per well), incubated for 6 hours at 37°C and 5% CO2, allowing them to adhere to the surface. Subsequently, they were treated with ADH-1 (at 0.25; 0.50 or 1 mg/ml) for 12, 24, or 48 hours. Cell viability was determined using the CellTiter-Glo Luminescent Cell Viability Assay (Promega, Madison, WI) according to the manufacturer's protocol and luminescence measured using the LUMIstar Optima plate reader (BMG Labtech, Offenburg, Germany). Effects on cell death were confirmed using flow cytometric Annexin V staining. Briefly, cells were resuspended in 200 µl Annexin V buffer (Bender MedSystems, Vienna, Austria), supplemented with 1 µl Annexin V-FITC (Bender MedSystems) and incubated during 15 minutes in the dark at room temperature. Another 100 µl Annexin V buffer was added, supplemented with 4 µl Propidium Iodide (PI) (Beckman Coulter, Woerden, The Netherlands). Analysis was performed on a Cytomics FC-500 flow cytometer (Beckman Coulter).

### RNA extraction

Total RNA was extracted from 5×10^6^ to 1×10^7^ cells using RNeasy mini columns (Qiagen, Venlo, The Netherlands) following the instructions of the manufacturer. The concentration and purity of the recovered RNA was determined measuring the optical density (OD) at 260 and 280 nm. RNA quality was defined using the Experion RQI value. All RNA was DNase treated to remove contaminating genomic DNA. Briefly, 1 µg of RNA was incubated with 1 µl of 10× DNase I reaction buffer and 1 µl of DNase I (Invitrogen, Merelbeke, Belgium) for 15 minutes at room temperature. DNase I was inactivated by adding 1 µl of 25 mM EDTA solution and heating 10 minutes at 65°C.

### Reverse transcription quantitative PCR

cDNA was generated using the iScript cDNA synthesis kit (Bio-Rad, Nazareth Eke, Belgium) according to the instructions of the manufacturer. Reactions were performed using 2 µg DNase I – treated RNA. After cDNA preparation, the samples were diluted 10-fold and stored at −80°C in 50 µl volumes. RT-qPCR reactions were carried out using MESA GREEN qPCR Mastermix Plus (Eurogentec, Seraing, Belgium). Briefly, 2 µl cDNA was added to 23 µl of PCR mix (6.25 µl Reaction Buffer, 0.25 µl Forward Primer (600 nM), 0.25 µl Reverse Primer (600 nM) and 16.25 µl milliQ water). Reactions were carried out on an iQ5 real-time qPCR machine (Bio-Rad). Relative expression values were calculated using the ΔCt-method. For normalization, the expression of at least three reference genes was combined to calculate a normalization factor [33]. RT-qPCR analysis performed on the SIOPEN cohort was performed as described in Vermeulen *et al.* [30] and analyzed using the qbasePLUS 1.4 software (http://www.qbaseplus.com). Primer sequences were as follows: Fwd-CDH2: TCCAGACCCCAATTCAATTAATATTAC; Rev-CDH2: AAAATCACCATTAAGCCGAGTGA and the reference genes: FWD-B2M: GAGTATGCCTGCCGTGTG; REV-B2M: AATCCAAATGCGGCATCT; FWD-SDHA: TGGGAACAAGAGGGCATCTG; REV-SDHA: CCACCACTGCATCAAATTCATG; FWD-UBC: ATTTGGGTCGCGGTTCTTG; REV-UBC: TGCCTTGACATTCTCGATGGT; FWD-HPRT1: TGACACTGGCAAAACAATGCA and REV-HPRT1: GGTCCTTTTCACCAGCAAGCT. Primer sequences were submitted to RTPrimerDB database (http://www.rtprimerdb.org).

### Tissue Microarray Analysis

For the establishment of the tissue microarray, three representative areas (0.6 mm diameter) were selected from H&E stained slides of 84 formalin-fixed and paraffin-embedded primary untreated NB tumors of which sufficient clinical data were available (Table 1). Each of the three cores were embedded into a paraffin array multiblock (for layout, see Figure S2). Sections of 1.5 µm thickness were cut and transferred to positively charged glass slides, dried overnight at 37°C, dewaxed, and rehydrated by routine methods. Immunohistochemistry was performed on three such slides, using a peroxidase-labeled mouse monoclonal anti-human N-cadherin antibody (clone 3B9, Invitrogen, Merelbeke, Belgium) and the 3-amino-9-ethylcarbazole (AEC) substrate. An additional slide served as negative control. The immunostainings were evaluated by one experienced pathologist (K.B). First, tissue spots were checked for their presence, completeness or possible artifacts and those meeting these qualitative criteria were chosen for reporting the staining results. Slides were scored for immunoreactive neuroblasts, gangliocytic differentiated cells and neuropil. The following semi/quantitative scoring system was used: no staining = 0; weak staining = 1; moderate staining = 2 and strong staining = 3 and applied on each of the three histological targets. In case of heterogeneous staining patterns, subpopulations of different staining intensity were taken into account by adding or subtracting (according to their intensity compared to the main population) 0.3 points from the initial value (since most of the subpopulations represented ∼30% of the immunoreactive cells). Subsequently these results were averaged over all graded histological targets in a spot and all triplicates. After six months, all spots were evaluated once more by the same observer, without knowledge of the first results. The final results used for statistical analysis represent average scores from both evaluations.

### Western blotting

Exponentially growing cells were harvested and lysed in lysis buffer (50 mM Tris-HCl, pH 7.5, 250 mM NaCl, 1% NP-40 (Sigma, Bornem, Belgium), 1% Triton X-100 (Sigma) and protease inhibitor cocktail tablets (Roche, Vilvoorde, Belgium)). Cleared lysates were assayed for protein concentration by using the Bio-Rad (Hemel Hempstead, Hertis, United Kingdom) protein assay system and subjected to immunoblotting. Bound primary antibodies were detected with horseradish peroxidase (HRP)–goat anti-mouse antibody (Amersham Pharmacia Biotech, Uppsala, Sweden). Enhanced chemiluminescence Western blotting detection reagents were purchased from Amersham. The following primary antibodies were used: a mouse monoclonal anti-human N-cadherin antibody (clone 3B9, Invitrogen, Merelbeke, Belgium) and a mouse anti-alpha tubulin (Sigma, 1/4000).

### Statistical analysis

Analysis of cell viability data was performed using GraphPad Prism software version 5.01 for Windows (GraphPad Software, San Diego, CA). All other statistics were performed in SPSS version 15 for Windows (SPSS, Chicago, IL). Study populations were described by standard descriptive statistics. Differences in expression (RNA, protein) between patient groups (non-metastatic vs. metastatic; high risk vs. low risk; favorable vs. unfavorable prognosis) were analyzed by the non-parametric Mann-Whitney U test. All statistical tests were two-sided. P-values less than 0.05 were considered statistically significant.

## Results

### N-cadherin expression in neuroblastoma cell lines

**Figure 1:**
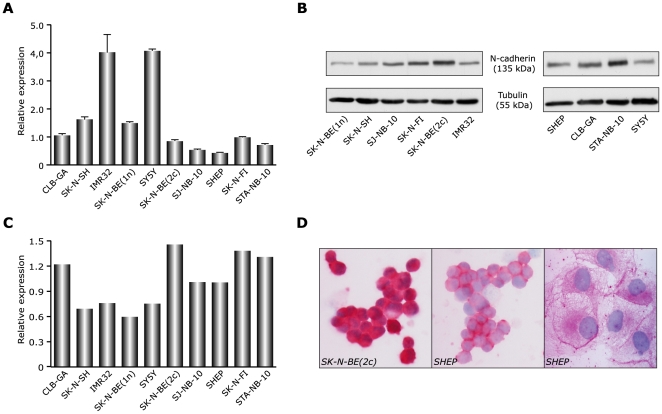
Expression of N-cadherin transcript in NB cell lines. A. gene expression was measured across a panel of 10 human NB-derived cell lines using quantitative RT-qPCR. Values were normalized to the B2M and UBC housekeeping genes. The ΔCt method was used for relative quantification. Data are mean ± standard deviation (n = 3). B. Western blot showing protein expression of N-cadherin across the 10 cell lines. α-tubulin was used as a loading control, shown below the N-cadherin blot. C. Relative quantification of N-cadherin protein expression. Protein expression was normalized to the α-tubulin loading control. D. Immunocytochemical staining of NB cell lines, confirming protein expression. Images taken at 100× magnification.

We first quantified the N-cadherin mRNA expression across a panel of 10 NB cell lines. Table S1 lists their general characteristics. Relative quantification of N-cadherin mRNA levels using RT-qPCR showed that all NB cell lines expressed N-cadherin (Fig. 1A), with the highest mRNA levels observed in IMR32 and SH-SY5Y cells. Gene expression data were confirmed by Western blot (Fig. 1B) and semi-quantitative analysis of the blots revealed that high protein expression was observed in SK-N-BE(2c), SK-N-FI, STA-NB-10 and CLB-GA cells (Fig. 1C). Immunocytochemical staining for N-cadherin further evidenced the N-cadherin expression in different NB cell lines at varying levels (Fig. 1D).

### N-cadherin expression correlates with metastatic disease

**Figure 2:**
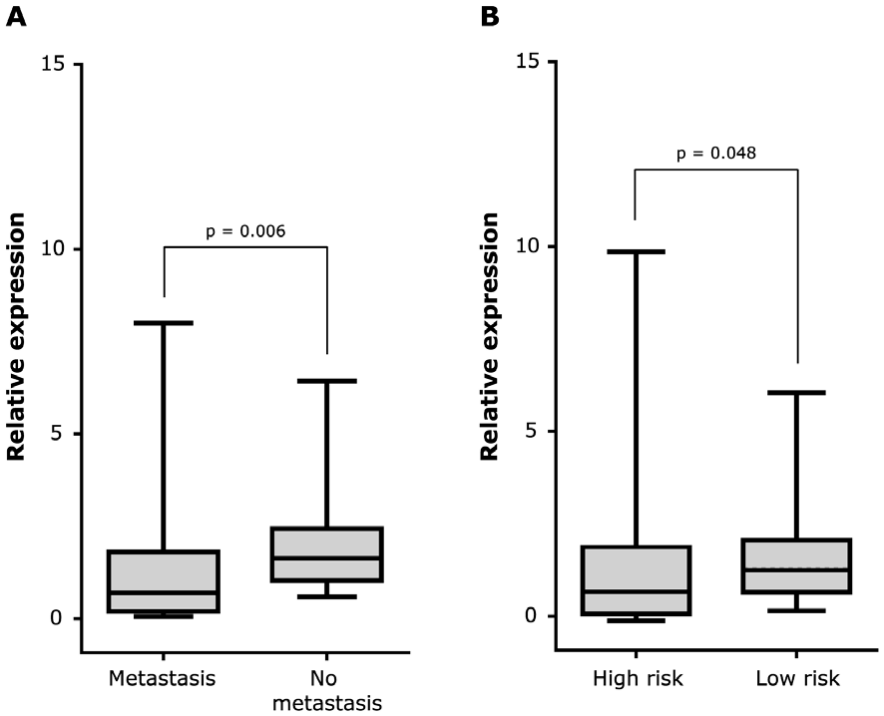
N-cadherin mRNA levels negatively correlate to metastasis and risk definition. A. N-cadherin mRNA expression and the presence/absence of metastasis at diagnosis. B. N-cadherin mRNA expression according to risk group (high risk vs. others). The median (horizontal line), the interquartile range (box), and the upper and lower range of the data (whiskers) are shown.

Next, we studied the N-cadherin mRNA expression in a sample-set of 356 neuroblastoma tumors. Table 1 shows the clinical details of the patients. According to the International Neuroblastoma Staging System (INSS), 77 patients were classified with a stage 4 tumor and 35 patients were diagnosed with stage 4S disease. Both stage 4 and 4S patients suffer from metastatic disease at diagnosis. By means of RT-qPCR, we detected N-cadherin mRNA expression in all 356 primary tumor samples (Figure S1), as was previously reported by Amitay and colleagues [34]. In an effort to associate N-cadherin expression with clinical parameters, the relation between N-cadherin expression and metastatic disease was evaluated. Since the N-cadherin mRNA expression was not normally distributed (Kolmogorov-Smirnov test, p<0.001), non-parametric statistical tests (Mann-Withney U, Kruskall-Wallis) were used. The median relative expression level was significantly lower in metastasized NB tumors (stage 4 and 4S) compared to localized tumors (stages 1, 2 and 3) (p = 0.006). Moreover, patients with high risk disease (stage 4>1 year and stage 1, 2, 3 and 4S patients with MYCN amplification) presented with a lower N-cadherin expression (p = 0.048) compared to low risk patients (Fig. 2A and B).

**Figure 3:**
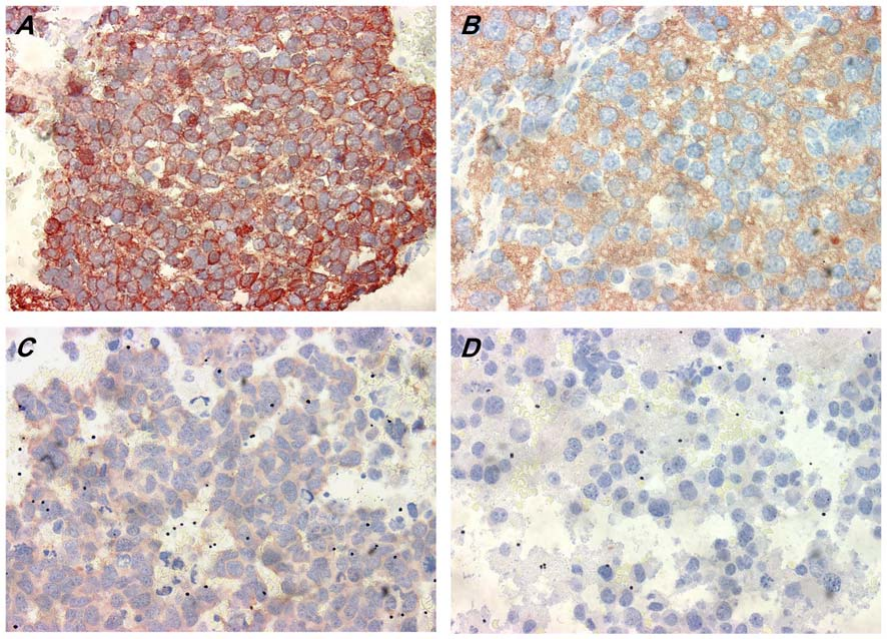
Immunohistochemical expression of N-cadherin on TMA. Immunohistochemical staining was performed and results scored as described in Materials and Methods. Images are representative for strong (a), moderate (b) to weak level (c) or, in one case, negative staining (d). Note: All four tumors are stained simultaneously on the same TMA slide facilitating a comparison of staining intensities. Images were taken with an ×60 dry lens and are presented at identical magnification.

**Figure 4:**
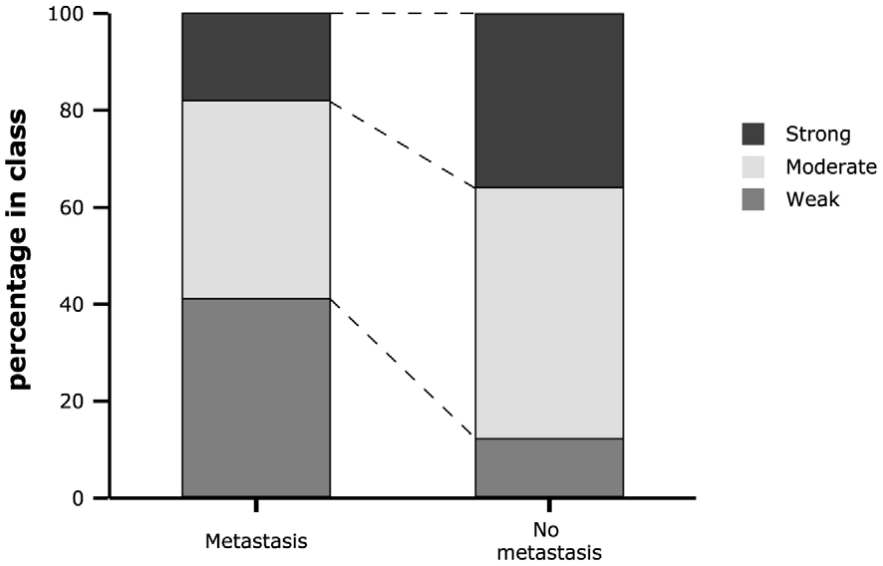
N-cadherin protein levels correlate with metastatic disease. Visualized is the distribution of samples (percentage) staining weak, moderate and strong in each class (metastasized N = 34, or non-metastasized tumors N = 39). The number of samples in each class was set to 100 percent.

Next, we investigated the observed reduced expression of N-cadherin in metastatic high risk NB at the protein level using a dedicated Tissue MicroArray (TMA). A total of 84 paraffin embedded tumors, of which sufficient clinical information was available, were selected for this purpose. The general characteristics of the 84 patients are summarized in Table 1. To obtain an adequate representation of the whole sections, 3 punches from each of the 84 tumors were embedded in the paraffin array block, followed by sectioning. A schematic view on the array is given in Figure S2. Three independent slices of the paraffin array block were stained for N-cadherin protein expression. In agreement with the RT-qPCR data, the N-cadherin protein was detected in all punches, albeit at different levels (Figure 3). We further classified the N-cadherin protein expression according to the quartile ranges of the expression data into weak, moderate and strong expression (see Materials and methods). For further analyses, we only took into account the primary tumor samples present on the TMA (N = 73). As observed for mRNA expression, N-cadherin protein expression was significantly lower in metastasized primary NB tumors compared to non-metastasized tumors (Fisher's Exact Test, p = 0.012) (Figure 4). Patients with high risk disease presented with a lower N-cadherin protein expression (p = 0.032). Interestingly, we observed that N-cadherin expression significantly correlated with the recorded INPC prognosis (favorable vs. unfavorable status), where low N-cadherin expression predicts an unfavorable status (p = 0.004).

### Functional inhibition of N-cadherin decreases cell viability

In order to study the functional role of N-cadherin in NB-cells, a N-cadherin antagonistic model was established by addition of ADH-1 to NB cell lines. ADH-1 inhibits N-cadherin mediated interactions. Moreover, ADH-1 is known to induce apoptosis and alter the intracellular distribution of β-catenin and actin in endothelial cells [26]–[29].

**Figure 5:**
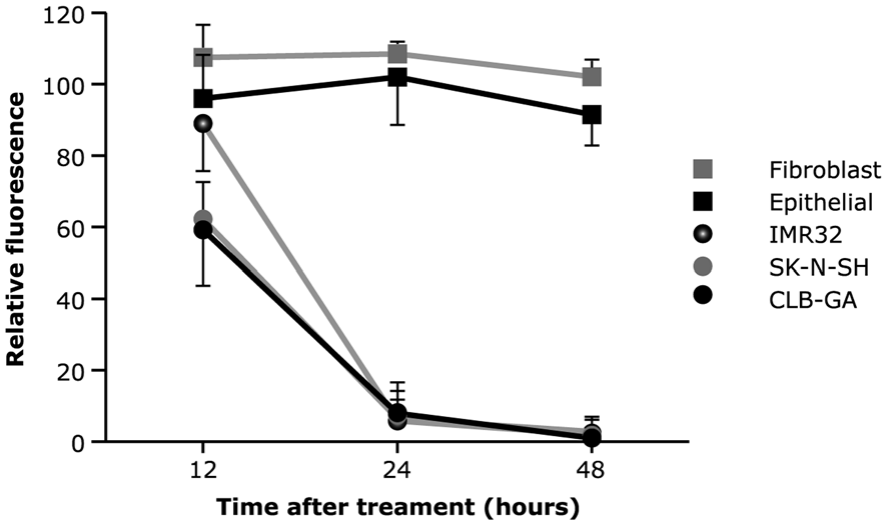
ADH-1 induces cell death in vitro. ADH-1 (1 mg/ml) was added to exponentially growing cultures of epithelial cells, fibroblasts and the indicated NB cell lines. Cell survival was measured at 12, 24 and 48 h after addition of ADH-1, using the CellTiter-Glo luminescent Cell Viability assay (Promega). Data were normalized to no drug treatment. Data are mean ± standard deviation (n = 3).

Erez *et al.* reported that ADH-1 induces apoptosis in endothelial cells when applied at concentrations greater than 0.25 mg/ml [35]. These data led us to examine the dose dependent effect of ADH-1 on NB cell lines (CLB-GA, IMR32, SK-N-SH), fibroblasts and epithelial cell cultures. The number of viable cells in culture was measured using the CellTiter-Glo luminescent Cell Viability assay (Promega) at 12 h, 24 h and 48 h after addition of ADH-1 (0.25 mg/ml; 0.50 mg/ml and 1 mg/ml). ADH-1 proved to be a potent inducer of cell death in NB cell lines when added at 1 mg/ml. At this concentration, more than 50% of NB cells underwent cell death 24 h after addition (Fig. 5). Interestingly, no effect was seen on the survival of fibroblasts and N-cadherin negative epithelial cells (Fig. 5). These observations were confirmed by flow cytometric determination of PI and FITC-Annexin V levels (Figure S3).

## Discussion

The acquisition of invasive and metastatic properties by cancer cells during tumor progression is intimately linked to changes in the expression of adhesion molecules regulating the interactions of cancer cells with the extracellular matrix (ECM) and neighboring cells [17]–[21]. At present, only little information on the importance of N-cadherin expression in neuroblastoma, is available.

In this study, we have shown that N-cadherin transcript and protein expression can be detected in all NB cell lines and NB tumor samples. However no clear correlation was observed between RNA and protein levels in the different cell lines. Lack of correlation between mRNA and protein levels is often observed and may result from various post-translational mechanisms.

The observed association between reduced N-cadherin expression and the presence of metastasis is in accordance with the biological role of N-cadherin in neural development, where the absence of N-cadherin expression is a pre-requisite for neural crest migration and its re-expression correlates with a migration stop.

Remarkably, metastatic outgrowth does not seem to require extensive alteration in N-cadherin expression levels. This is in line with the finding of others, demonstrating that the metastatic process is not accompanied by major changes in gene expression [3], [36]. Nevo and colleagues recently reported 112 genes differentially expressed between metastatic and non-metastatic NB cells, using an orthotopic model of a human NB local and metastatic variant having the same genetic background [3]. Unfortunately, the N-cadherin gene was not represented in their set of differential genes, possibly due to differences in experimental setup and data threshold cut off values.

Classical cadherins regulate, amongst others, apoptotic processes. For example, N-cadherin-mediated cell adhesion plays a pivotal role in follicular and luteal cell survival and its disruption induces apoptosis of these cells in culture [37]–[39]. Similarly, it was demonstrated that disruption of cadherin-mediated adhesion in mouse intestinal epithelial cells leads to cell death [40]. In our hands, the addition of 1 mg/ml ADH-1 peptide caused significant apoptotic cell death in all NB cell lines investigated, which was not seen in N-cadherin negative epithelial cells, neither in fibroblasts. Moreover, the detection of phosphatidylserine strongly suggests that the detected cell death is mainly due to apoptosis. The strong negative effect on cell viability at this clinically feasible dose, combined with the expression of N-cadherin in all NB samples, suggest N-cadherin as a potential novel therapeutic target in the treatment of NB disease. Moreover, to our knowledge, no N-cadherin gene deletions, amplifications or mutations, possibly hampering N-cadherin-based treatment of NB, are described, offering a broad therapeutic application domain. However, this needs further investigation, including the effect on cell viability of all other signaling molecules in the N-cadherin pathway. In addition, recent clinical trials using ADH-1 report low toxicity and antitumor activity in a variety of cancers including esophageal, non-small cell lung, renal cell and hepatocellular carcinomas [26]–[29]. While preparing this manuscript, Adherex set off a screen for small molecule N-cadherin antagonists, principally having structural similarities to the HAV region of N-cadherin. Burden-Gulley and colleagues demonstrated that those novel compounds are even more potent than ADH-1 at perturbing N-cadherin-mediated neurite outgrowth and cell adhesion [41]. Concerning their higher potency, and their longer half-life compared to the ADH-1 peptide, it will be of high interest to evaluate their impact on N-cadherin mediated apoptotic cell death in NB cells.

At first glance, it may seem paradoxical that low N-cadherin levels correlate with metastatic disease, and on the other hand inhibition of N-cadherin signaling results in apoptotic cell death. However, the in vitro experiments clearly indicate that neuroblastoma cells are sensitive to apoptosis, here stimulated by ADH-1, an inhibitor of N-cadherin. This however also could implicate that cells which do harbor only low levels of N-cadherin are less sensitive to apoptosis induction, allowing them to circumvents signals of death provoked by losing contact signals. In other words, together our experiments could indicate that, low expression of N-cadherin implicates a higher apoptosis resistance and with this the possibility of metastasis.

In summary, we have demonstrated that low N-cadherin expression strongly correlates with the metastatic dissemination of NB tumors. Importantly, our functional experiments showed that the N-cadherin antagonist ADH-1 has significant antitumor activity against neuroblastoma cells *in vitro*. Since virtually all neuroblastoma samples express N-cadherin, the cell adhesion protein might be a valid target for treatment.

## Supporting Information

Figure S1 **Real-time RT-qPCR analysis of N-cadherin expression in 356 NB patients.** Gene expression was measured across a panel of 356 human NB samples using quantitative real-time RT-PCR. Values were normalized to the B2M, SDHA and HPRT1 housekeeping genes. The ΔCt method was used for relative quantification.
(TIF)

Figure S2 **Overview of tissue distribution on the TMA.**
(TIF)

Figure S3 **ADH-1 induces cell death in vitro.** ADH-1 (1 mg/ml) was added to exponentially growing cultures of fibroblasts and the indicated NB cell lines. Cell survival was measured at 12, 24 and 48 h after addition of ADH-1, using PI and Annexin V staining. Data were normalized to no drug treatment. Data are mean ± standard deviation (n = 3).
(TIF)

Table S1 **NB cell line characteristics.** *For immunophenotyping, values indicate percentage of cells stained. **DEL = deletion, AMP = amplification
(DOC)
