# Supplementary figures and images for: N-Cadherin in Neuroblastoma Disease: Expression and Clinical Significance
Source: PLoS One. 2012 Feb 15;7(2):e31206. doi: 10.1371/journal.pone.0031206 (PMC3280274; doi:10.1371/journal.pone.0031206)

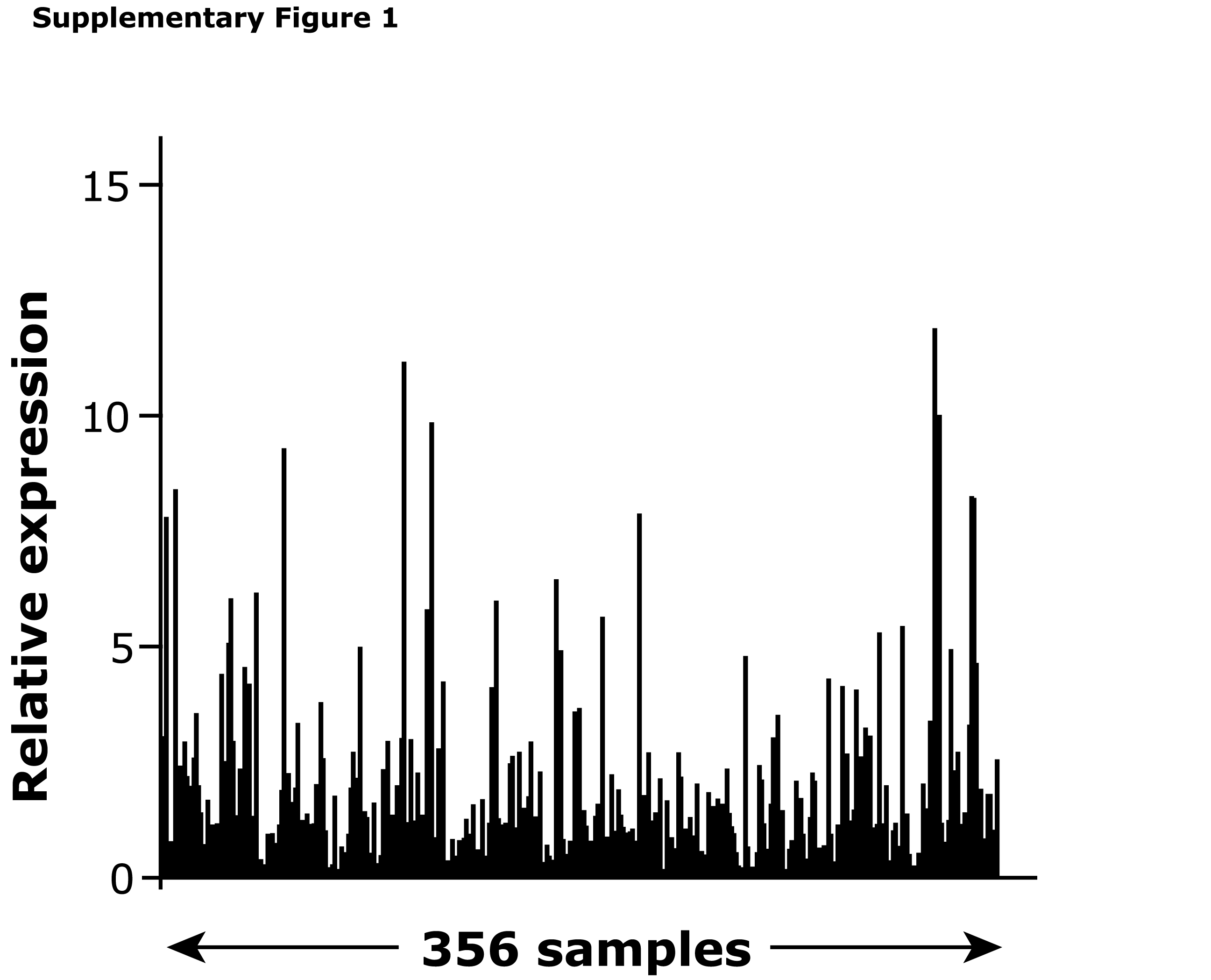

Supplement: Figure S1 — Real-time RT-qPCR analysis of N-cadherin expression in 356 NB patients. Gene expression was measured across a panel of 356 human NB samples using quantitative real-time RT-PCR. Values were normalized to the B2M, SDHA and HPRT1 housekeeping genes. The ΔCt method was used for relative quantification. (TIF) [file pone.0031206.s001.tif]

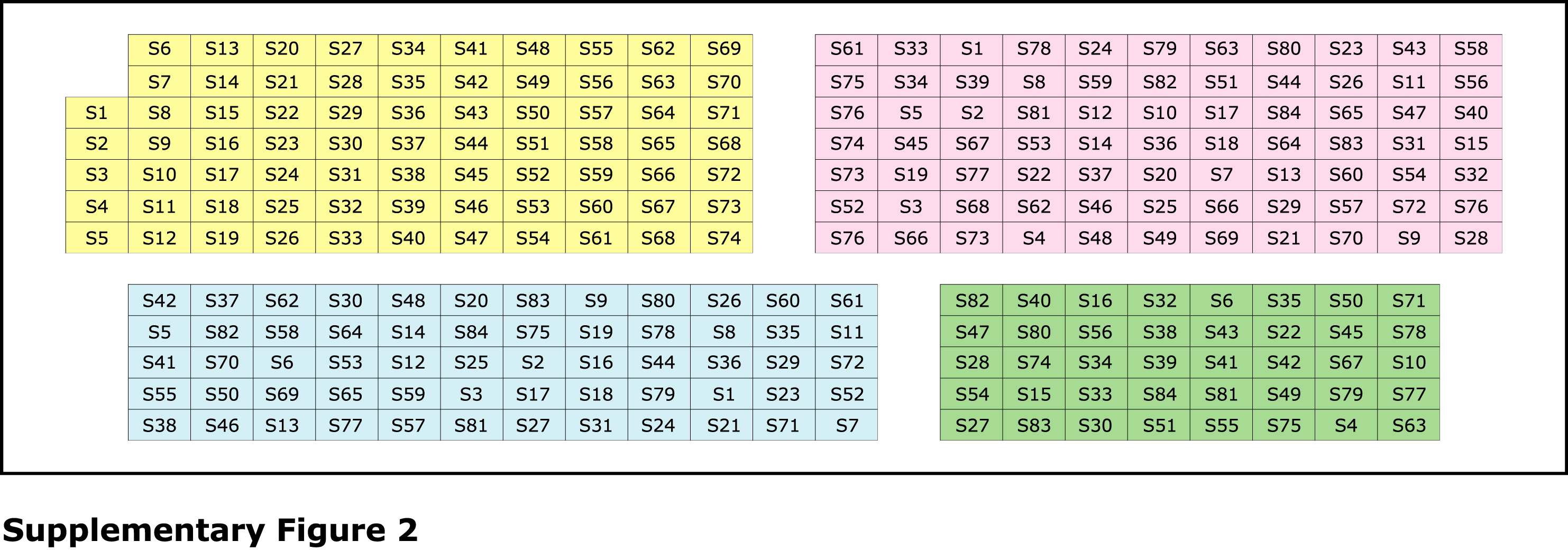

Supplement: Figure S2 — Overview of tissue distribution on the TMA. (TIF) [file pone.0031206.s002.tif]

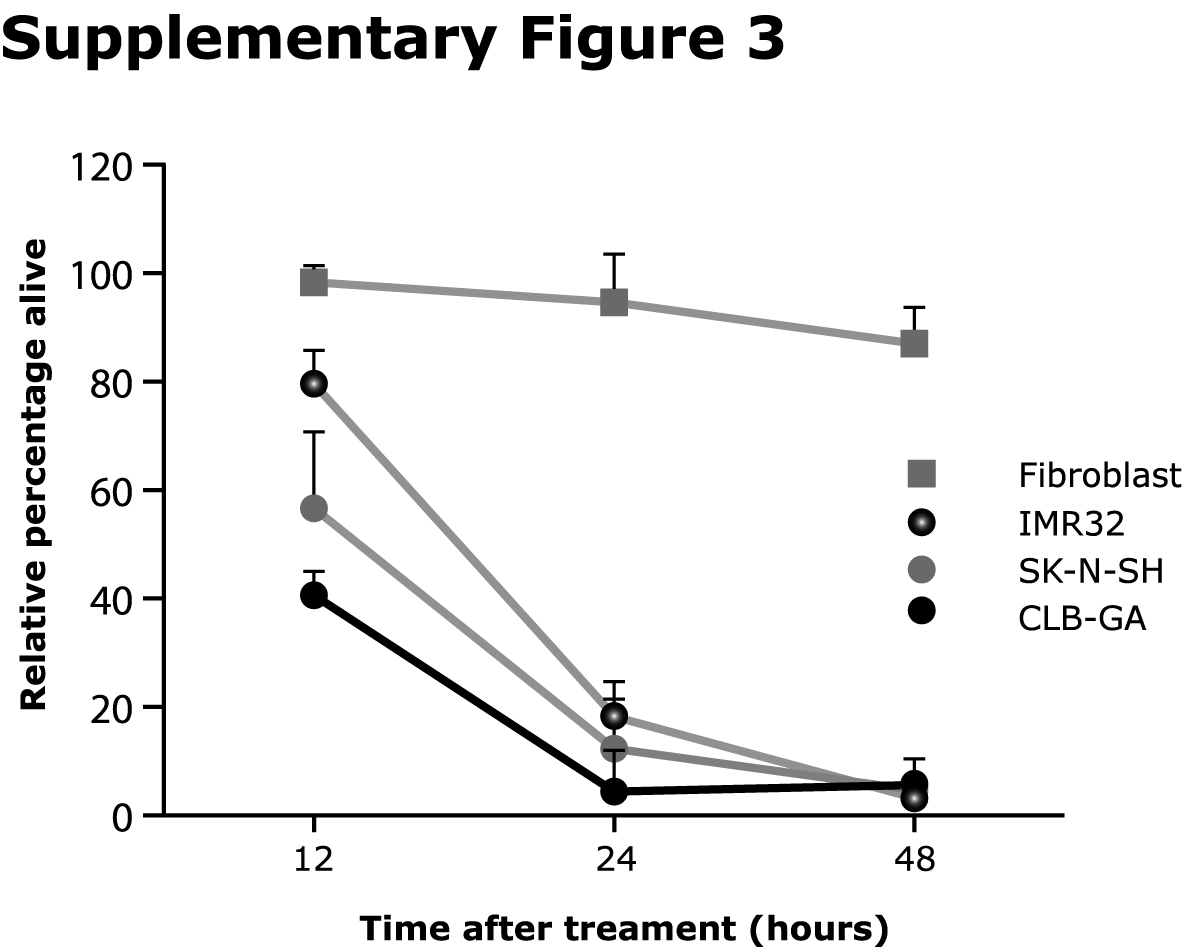

Supplement: Figure S3 — ADH-1 induces cell death in vitro. ADH-1 (1 mg/ml) was added to exponentially growing cultures of fibroblasts and the indicated NB cell lines. Cell survival was measured at 12, 24 and 48 h after addition of ADH-1, using PI and Annexin V staining. Data were normalized to no drug treatment. Data are mean ± standard deviation (n = 3). (TIF) [file pone.0031206.s003.tif]
