# Supplementary material for: N-Cadherin in Neuroblastoma Disease: Expression and Clinical Significance
Source: PLoS One. 2012 Feb 15;7(2):e31206. doi: 10.1371/journal.pone.0031206 (PMC3280274; doi:10.1371/journal.pone.0031206)
Supplement: Table S1 — NB cell line characteristics. *For immunophenotyping, values indicate percentage of cells stained. **DEL = deletion, AMP = amplification (DOC) [file pone.0031206.s004.doc]

**Supplementary Table 1 NB cell line characteristics**

|  | **Genetics** | | | | | | **Immunophenotyping** | | | **Reference** |
| --- | --- | --- | --- | --- | --- | --- | --- | --- | --- | --- |
| **Cell line** | **MYCN** | **1p** | **3p** | **11q** | **14q** | **17q** | **GD2** | **Vimentin** | **Neurofilament** |
| CLB-GA | - | DEL | DEL | DEL | - | GAIN | 90 | <1 | <1 | Combaret et al. (1995) |
| SY5Y | - | - | - | - | - | - | 90 | 20 | 50 | Biedler et al. (1973) |
| SK-N-SH | - | - | - | - | - | GAIN | na | na | na | Biedler et al. (1973) |
| SHEP | - | - | - | - | - | GAIN | 5 | 95 | <1 | Biedler et al. (1973) |
| SK-N-BE (1n) | AMP | DEL | - | - | - | - | 5 | 55 | 85 | Biedler et al. (1978) |
| SK-N-BE (2c) | AMP | DEL | DEL | - | - | - | 5 | 50 | 85 | Barnes et al. (1981) |
| IMR32 | AMP | DEL | - | DEL | - | GAIN | 30 | 60 | 70 | Tumilowizc et al. (1970) |
| SK-N-FI | - | - | - | - | - | - | na | na | na | Ciccarone et al. (1989) |
| SJ-NB-10 | AMP | DEL | DEL | - | - | GAIN | 30 | 75 | 60 | Shapiro et al. (1993) |
| STA-NB-10 | AMP | DEL | - | - | - | - | 5 | 50 | 60 | Ambros et al. (1997) |

*For immunophenotyping, values indicate percentages of cells stained

*DEL=deletion, AMP=amplification

Ambros IM, Rumpler S, Luegmayr A, Hattinger CM, Strehl S, et al. (1997) Neuroblastoma cells can actively eliminate supernumerary MYCN copies by micronucleus formation—sign of tumour cell revertance? *Eur J Cancer* 33: 2043-2049.

Barnes EN, Biedler JL, Spengler BA, Lyser KM (1981) The fine structure of continuous human neuroblastoma Lines SK-N-SH, SK-N-BE(2), and SK-N-MC. *In Vitro* 17: 619-631.

Biedler JL, Helson L, Spengler BA (1973) Morphology and growth, tumorigenicity, and cytogenetics of human neuroblastoma cells in continuous culture. *Cancer Res* 33: 2643-2652.

Biedler JL, Roffler-Tarlov S, Schachner M, Freedman LS (1978) Multiple neurotransmitter synthesis by human neuroblastoma cell lines and clones. *Cancer Res* 38: 3751-3757.

Ciccarone V, Spengler BA, Meyers MB, Biedler JL, Ross RA (1989) Phenotypic diversification in human neuroblastoma cells: expression of distinct neural crest lineages. *Cancer Res* 49: 219-225.

Combaret V, Turc-Carel C, Thiesse P, Rebillard AC, Frappaz D et al. (1995) Sensitive detection of numerical and structural aberrations of chromosome 1 in neuroblastoma by interphase fluorescence in situ hybridization. Comparison with restriction fragment length polymorphism and conventional cytogenetic analyses. *Int J Cancer* 61: 185-191.

Shapiro DN, Valentine MB, Rowe ST, Sinclair AE, Sublett JE, et al. (1993) Detection of N-myc gene amplification by fluorescence in situ hybridization. Diagnostic utility for neuroblastoma. *Am J Pathol* 142: 1339-1346.

Tumilowicz JJ, Nichols WW, Cholon JJ, Greene AE (1970) Definition of a continuous human cell line derived from neuroblastoma. *Cancer Res* 30: 2110-2118.
